# Multimodal Interventions Are More Effective in Improving Core Symptoms in Children With ADHD

**DOI:** 10.3389/fpsyt.2021.759315

**Published:** 2021-12-16

**Authors:** Ke Ning, Tingzhao Wang

**Affiliations:** ^1^Physical Education Institute of Shaanxi Normal University, Xi'an, China; ^2^School of Education Shaanxi Normal University, Xi'an, China

**Keywords:** children with ADHD, core symptoms, multimodal interventions, sensory integration training, EEG biofeedback therapy

## Abstract

**Objective:** To investigate the effect of sensory integration training combined with EEG biofeedback on core symptoms in children with ADHD.

**Methods:** Fifty-two children with attention-deficit, hyperactive-impulsive and combined ADHD were selected. They were randomly divided into control group, sensory integration training group, EEG biofeedback group, and sensory integration training + EEG biofeedback group, and after 4 months of intervention, concentration time and impulsive- hyperactivity and hyperactivity index scores on the PSQ scale were assessed.

**Results:** Compared with that before the intervention, the attention time was significantly increased (*P* < 0.01), and the impulsive-hyperactivity and hyperactivity index scores were significantly decreased (*P* < 0.05, *P* < 0.01). After the intervention, the attention time was significantly higher than that of the control group (*P* < 0.05, *P* < 0.01), the attention time of the multimodal intervention group was significantly higher than that of the single intervention group (*P* < 0.01), and the impulsive-hyperactivity and hyperactivity index scores were significantly lower than those of the single intervention group (*P* < 0.05).

**Conclusion:** Multimodal intervention can significantly improve the concentration level of children with ADHD, and significantly improve the behavioral symptoms of impulsive-hyperactivity and hyperactivity. Multimodal interventions were more effective than single interventions in improving core symptoms in children with ADHD. The results of this study provide a reference for related research and practical application.

## Impact

- Sensory integration training combined with EEG biofeedback therapy multimodal intervention is more effective than single intervention in improving core symptoms in children with ADHD.
- To provide theoretical support for multimodal non-pharmacological multimodal intervention to improve core symptoms in children with ADHD.
- Provide the basis for the development of multimodal intervention program for children with ADHD.

## Introduction

Attention deficit hyperactivity disorder (ADHD) is the most common neurobehavioral disorder in childhood and adolescence (1). ADHD is usually characterized by a wide range of symptoms of inattention and transient attention, hyperactivity, and impulsivity disorders, which will cause severe impairment to their academic and daily life activities (2). The results of epidemiological surveys indicate that about 5% of children and adolescents and 2.5% of adults worldwide are affected by the disease (3). According to statistics, the prevalence of ADHD in Chinese children is about 6.3%, the number of children is more than 23 million, and the prevalence is on the rise in recent years. The pathogenesis of the disease has not been clarified, and multiple causes such as environmental and genetic risk factors appear to be complex etiologies of ADHD (4). Many children with ADHD have complications such as lack of self-esteem, interpersonal conflict, poor academic performance, and increased risk of substance abuse during adolescence, which may adversely affect their physical and mental health and family life and social skills in adulthood (5). According to the criteria of the Diagnostic and Statistical Manual of Mental Disorders, ADHD can be divided into three categories: attention-deficit, hyperactive/impulse-control disorder, and combined (6). Studies have shown that the core symptoms of ADHD include inattention, hyperactivity and impulsiveness (7). At present, the main treatments for ADHD are drug therapy and non-drug therapy (mainly including behavioral therapy and cognitive behavioral therapy) and combination therapy, with the purpose of improving the core symptoms of children with ADHD and reducing behavioral problems. In most situations and guidelines, pharmacological treatment remains the mainstay of treatment for ADHD, with psychostimulant medication being the most widely used drug to treat symptoms in children with ADHD (8–10). The effectiveness of psychostimulants in the treatment of symptoms in children with ADHD has been well-documented in a large body of literature, significantly improving the function of attention, impulsivity and socio-behavioral symptoms in children with ADHD (11–13). However, psychostimulant therapy also has some limitations, such as the short duration of treatment, a significant proportion (about 1/3) of children do not respond to psychostimulant therapy and/or are intolerant (14, 15), and is accompanied by short-term adverse effects, such as fatigue, nausea and vomiting and loss of appetite (16). Although the long-term side effects of drug therapy have not been determined, it is increasingly recognized that continuous drug therapy may have adverse cardiovascular effects in children and may affect the growth and development of children (17). To cope with these deficiencies, researchers are increasingly focusing on and seeking non-pharmacological alternative treatment strategies in addition to drugs. At the same time, parents of children with ADHD are also increasingly inclined to choose non-drug therapies (18). There is strong evidence that pharmacological treatment and non-pharmacological interventions such as psychoeducation programs, behavioral interventions, and cognitive behavioral therapy have a major beneficial effect on the core symptoms of ADHD in ~80% of cases, at least in the short term (19–24). Sensory integration refers to the neuropsychological process in which the brain unifies individuals after inputting information from different sensory pathways such as vision, hearing, touch, and sniffing (25). Sensory integration training refers to a training method that uses the plasticity of neurological system during ontogeny to make a correct adaptive response through multimodal training in spatial perception, balance sensation, and vision and hearing. Its core content is sports games, which can improve the symptoms of children in terms of inattention, hyperactivity or impulsivity, with relatively low difficulty of operation, treatment cost, and the advantages of teaching in music (26, 27). EEG biofeedback technology (also known as neurofeedback) is to extract specific parameters in EEG signals as monitoring indicators, and use these parameters for brain function training, in order to achieve the purpose of treating diseases and functional rehabilitation. With the advantages of non-invasiveness and less side effects, EEG biofeedback technology has gradually become an effective treatment for cognitive behavioral dysfunction diseases such as ADHD and autism (28, 29). However, there are few reports on the effect of sensory integration training combined with EEG biofeedback multimodal intervention on the core symptoms of children with different types of attention deficit hyperactivity disorder. This study intends to use sensory integration training combined with EEG biofeedback therapy multimodal intervention method, to explore its intervention effect on the core symptoms of children with different types of ADHD, and to provide the basis for the development of multimodal intervention program for children with ADHD.

## Materials and Methods

### Study Subjects

A total of 156 children with ADHD who met the study criteria from July 2019 to March 2020 at Xi'an Jiaotong University Children's Hospital were selected as the study subjects. Among them, 52 cases were attention-deficit type, 52 cases were hyperactive-impulsive type and 52 cases were combined type.

### Inclusion and Exclusion Criteria

#### Inclusion Criteria

Meet the diagnostic criteria for ADHD in children in the Diagnostic and Statistical Manual of Mental Disorders (5th Edition) (DSM-V) published by the American Psychiatric Association (30); IQ rating of Wechsler Intelligence Test in children > 70 (31).

#### Exclusion Criteria

Mental disorders other than ADHD, such as intellectual disability, widespread developmental disorders, affective disorders, autism spectrum disorders, tic disorders, cardiovascular diseases, epilepsy, and other organic brain diseases or comorbidities, were excluded.

All cases were first diagnosed children and did not receive any treatment before enrollment. This study was reviewed and approved by the ethics committee of the hospital. Explain the study methods, process and other specific circumstances to all children and parents, follow the voluntary principle, and sign the informed consent form.

### Study Methods

According to the date and order of presentation of the children, random sampling was achieved using an SPSS random number generator. Children in the control group received 30 min of ADHD-related knowledge education only by their parents in the outpatient clinic. On the basis of the control group, the sensory integration training intervention group was given sensory integration training intervention, the EEG biofeedback group was given EEG biofeedback treatment, and the multimodal group was also given sensory integration training intervention and EEG biofeedback treatment for 4 months, as follows.

### EEG Biofeedback Therapy

VBFB300 brain biofeedback therapy system (Nanjing Weisi Medical Technology Co., Ltd.) was used to set the inhibition of 4–8 Hz theta wave and strengthen the sensorimotor rhythm of 12–16 Hz as the treatment target. Baseline tests were performed before treatment and again after the end of treatment. Through the acquisition of the child's brain waves and real-time feedback in the form of various images. Each treatment includes five segments, of which the first segment is the basic state detection and training goal formulation; the remaining four segments are the feedback treatment stage. 30 min/time, 5 times/week, for 4 months.

### Sensory Integration Training Intervention

According to the actual situation of the child, a personalized training program was developed. The training was guided by a professionally trained physician. The training content is mainly aimed at improving vestibular balance, proprioception, touch, intrinsic balance and learning ability and other training activities. The training equipment includes slide, skateboard climbing, stick insertion, jumping bed, sheep corner ball, balance table, cylinder cable, round horse bucket, kangaroo jumping, unicorn chair, four-corner shaking balance plate, lying ball, sunshine tunnel, circular trolley, tactile ball, S-shaped balance beam, etc. 5 times/week, 60 min/time for 4 months. Training principles: compliance, internal drive, happiness, taking children as the protagonist, cultivating confidence, pertinence, interest and step-by-step principles. Change the use method of various equipment, use various postures such as sitting, lying, standing, jumping, shaking, and rolling, make full use of various equipment, and train the sensory integration ability of children in a targeted manner.

### Evaluation Indicators and Quality Control

Concentration time: The concentration time of the children in a quiet and independent state was recorded before and after treatment. In a special room, the child was given a pile of building blocks to play building blocks at will in a quiet and independent state, and the length of time the child focused on continuously playing building blocks was recorded.

Conners ParentSymptom Questionnaire (PSQ) (32): This scale is a commonly used assessment scale that reflects hyperactivity and behavioral aspects in children with ADHD, i.e., The PSQ contains 48 items and, in addition to a total score, there are six subscale scores: Conduct problem (To be rude to an adult; Excessive temper; Destructive; Repudiate mistakes or convict others; Quarrel; Pout and anger; Stealing; Disobedient or barely obeying; Bullying others; Rapid and intense mood change; Discipline or restraint not preferred or followed; It's a child who's emotionally unhappy), Learning problem (Learning difficulties; Doing things has a good start, but a bad ending; Easy distraction or lack of concentration; Easy to deflate), Psychosomatic disorders [Headache; Stomachache; Other pain; Vomiting or nausea; Have problems passing stools (diarrhea, irregularity, and stool density)], Hyperactivity/ Impulsivity (excitable, impulsive; gesticulate; body twists constantly; restlessness, and often busy), Anxiety [Fear (new environment, strangers, strange places, and school); Shyness; Worry more than others (worry, loneliness, illness, and death); Let yourself be deceived], and Hyperactivity index (excitable, impulsive; Easy or frequent crying; body twists constantly; Restlessness, often “busy;” Destructive; Doing things has a good start, but a bad ending; Easy distraction or lack of concentration; Rapid and intense mood change; Easy to deflate; Nuisance to other children). Using the 4-level scoring method (0 = not at all, 1 = a little, 2 = much, 3 = very much), the more prominent the behavioral problems, the reliability and validity have been widely tested. This study was assessed before and after treatment, and the evaluation indicators were impulsive-hyperactivity and hyperactivity indices.

Quality control: All intervention modalities were performed under the guidance of a trained physician. Adequately communicate with parents and children before implementing the intervention and develop an individualized intervention program. The EEG biofeedback therapist recorded the child's performance during treatment in detail and fed back to the treating physician in a timely manner. Parents were followed up in the outpatient clinic at a fixed time every week for about 30 min each time to understand the implementation of the family intervention program and the child's performance, and inappropriate communication and training methods were adjusted in a timely manner. Children who were unable to adhere to sensory integration training or biofeedback therapy were withdrawn from this study.

### Statistical Analysis

Statistical analysis was performed using SPSS 23.0. Enumeration data were expressed as constituent ratio (%), and χ^2^test was used for comparison. Measurement data were expressed as mean ± standard deviation ($\bar{\chi }$ ± s). Paired *t*-test was used for comparison before and after treatment. Analysis of variance was used for comparison between multiple groups. Tukey method was used for pairwise comparison. *P* < 0.05 was considered statistically significant.

## Results

### General Information

A total of 156 children with ADHD [male, *n* =118 (75.6%); female, *n* = 38 (24.4%)] with a mean age of 7.7 ± 0.69 years were collected in this study. There was no significant difference in age or gender distribution among the four groups of children with ADHD (*P* > 0.05) (Table 1).

**Table 1:** Age and gender distribution of children with ADHD in the four groups.

**Groups**	**Number**	**Sex [*****n*** **(%)]**		**Year**
		**M**	**F**	**($\bar{\chi }$ ± S)**
CON	39	28 (72)	11 (28)	7.78 ± 0.6
SIT	39	30 (77)	9 (23)	7.9 ± 0.6
EEG	39	31 (80)	8 (20)	7.7 ± 0.5
MUL	39	29 (74)	10 (26)	8.0 ± 0.7
χ^2^/F		1.813		1.969
*P*		0.612		0.121

*CON, control group; SIT, sensory integration training group; EEG, electroencephalo -gram biofeedback group; MUL, multimodal intervention group. M, male; F, female*.

### Attention Span

Before the intervention, there were no significant differences in concentration time among the three types of ADHD children (*P* > 0.05). Compared within the post-intervention group, the results showed that the concentration time of children with ADHD in the three types of different intervention groups was significantly increased after intervention with different intervention methods compared with that before intervention (*P* < 0.01). After the intervention, the results showed that the concentration time of ADHD children in the three types of different intervention groups was significantly higher than that in the control group (*P* < 0.01, *P* < 0.05), and the concentration time of children with the three types of ADHD in the multimodal intervention group was significantly higher than that in the sensory integration training intervention group (*P* < 0.05, *P* < 0.01) (Tables 2–4).

**Table 2:** Comparison of concentration time before and after intervention in children with attention deficit disorder ($\bar{\chi }$ ± S, min).

**Groups**	**Number**	**Pre-intervention**	**Post-intervention**	***t***	***P***
CON	13	6.69 ± 0.79	6.75 ± 0.75	−0.2	0.85
SIT	13	6.54 ± 0.56	10.13 ± 1.81^**^	−6.48	0.00
EEG	13	6.44 ± 0.96	9.42 ± 1.38^**^	−9.92	0.00
MUL	13	6.76 ± 0.99	11.77 ± 1.74^**^,^&&^, ^##^	−8.93	0.00
*F*		0.40	27.93		
*P*		0.75	0.000		

*CON, control group; SIT, sensory integration training group; EEG, electroe ncephalo- gram biofeedback group; MUL, multimodal intervention group.*

*Compared with the control group,*

** *P < 0.01; compared with the psycho-behavioral group,*

&& *P < 0.05; compared with the biofeedback group,*

## *P < 0.01*.

**Table 3:** Comparison of concentration time before and after intervention in hyperactive-impulsive children ($\bar{\chi }$ ± S, min).

**Groups**	**Number**	**Pre-intervention**	**Post-intervention**	***t***	***P***
CON	13	7.07 ± 0.9	6.98 ± 1.01	1.02	0.33
SIT	13	7.29 ± 1.13	9.47 ± 0.99^**^	−5.58	0.00
EEG	13	7.30 ± 1.04	9.20 ± 0.64^**^	−16.26	0.00
MUL	13	7.04 ± 1.02	11.24 ± 1.51^**^,^&&^, ^##^	−13.83	0.00
*F*		0.24	26.46		
*P*		0.87	0.000		

*CON, control group; SIT, sensory integration training group; EEG, electroencephalo -gram biofeedback group; MUL, multimodal intervention group.*

*Compared with the control group,*

** *P < 0.01; compared with the psycho-behavioral group,*

&& *P < 0.01; compared with the biofeedback group,*

## *P < 0.01*.

**Table 4:** Comparison of concentration time before and after intervention in children with combined type ($\bar{\chi }$ ± S, min).

**Groups**	**Number**	**Pre-intervention**	**Post-intervention**	***t***	***P***
CON	13	7.38 ± 2.39	7.27 ± 2.28	0.80	0.44
SIT	13	7.64 ± 2.66	9.23 ± 2.94^*^	−3.12	0.01
EEG	13	7.39 ± 2.33	8.90 ± 2.47^*^	−6.56	0.00
MUL	13	8.11 ± 2.73	14.02 ± 3.36^**^,^&&^,^##^	−8.75	0.00
*F*		0.26	15.18		
*P*		0.86	0.00		

*CON, control group; SIT, sensory integration training group; EEG, electroencephalogram biofeedback group; MUL, multimodal intervention group.*

*Compared with the control group,*

* *P < 0.05,*

** *P < 0.01; compared with the psycho- behavioral group,*

&& *P < 0.01; compared with the biofeedback group,*

## *P < 0.01*.

### Impulsive-Hyperactivity and Hyperactivity Index Scores on the PSQ Scale

Before the intervention, there were no significant differences in impulsive-hyperactivity and hyperactivity index scores among different intervention groups among the three types of children (*P* > 0.05). After the intervention, the scores of impulsive-hyperactivity and hyperactivity index in different intervention groups were significantly lower in the three types of children compared with those before the intervention (*P* < 0.05, *P* < 0.01). The results showed that the scores of impulsive-hyperactivity and hyperactivity index in the multimodal intervention group of the three types of children were significantly lower than those in the control group, sensory integration training group, and EEG biofeedback therapy group (*P* < 0.05, *P* < 0.01) (Tables 5–10).

**Table 5:** Comparison of impulsive-hyperactivity scores before and after treatment in children with attention deficit disorder.

**Groups**	**Number**	**Impulsive-hyperactive**			
		**Pre-intervention**	**Post-intervention**	***t***	***P***
CON	13	1.51 ± 0.68	1.66 ± 0.61	−1.51	0.155
SIT	13	1.68 ± 0.59	1.58 ± 0.58	3.07	0.01
EEG	13	1.60 ± 0.44	1.49 ± 0.45	2.77	0.02
MUL	13	1.65 ± 0.59	1.36 ± 0.54^*^,^&^,^#^	5.67	0.00
*F*		0.22	2.39		
*P*		0.89	0.08		

*CON, control group; SIT, sensory integration training group; EEG, electroencephalogram biofeedback group; MUL, multimodal intervention group.*

*Compared with the control group,*

* *P < 0.05; compared with the psycho-behavioral group,*

& *P < 0.05; compared with the biofeedback group,*

# *P < 0.05*.

**Table 6:** Comparison of hyperactivity index scores before and after treatment in children with attention deficit disorder.

**Groups**	**Number**	**Hyperactivity index**			
		**Pre-intervention**	**Post-intervention**	***t***	***P***
CON	13	1.49 ± 0.62	1.62 ± 0.74	−1.67	0.12
SIT	13	1.62 ± 0.67	1.29 ± 0.59	2.31	0.04
EEG	13	1.64 ± 0.53	1.40 ± 0.38	3.88	0.002
MUL	13	1.46 ± 0.63	1.21 ± 0.61^**^,^&^,^#^	4.73	0.001
*F*		0.31	5.36		
*P*		0.82	0.003		

*CON, control group; SIT, sensory integration training group; EEG, electroencephalogram biofeedback group; MUL, multimodal intervention group.*

*Compared with the control group,*

** *P < 0.01; compared with the psycho-behavioral group,*

& *P < 0.05; compared with the biofeedback group,*

# *P < 0.05*.

**Table 7:** Comparison of impulsive-hyperactivity scores before and after treatment in children with hyperactive-impulsive type.

**Groups**	**Number**	**Impulsive-hyperactive**			
		**Pre-intervention**	**Post-intervention**	***t***	***P***
CON	13	1.41 ± 0.47	1.52 ± 0.39	−1.23	0.24
SIT	13	1.74 ± 0.69	1.53 ± 0.63	2.70	0.02
EEG	13	1.75 ± 0.55	1.61 ± 0.63	1.5	0.16
MUL	13	1.66 ± 0.64	1.02 ± 0.16^*^,^&^,^#^	4.5	0.00
*F*		0.94	5.64		
*P*		0.43	0.01		

*CON, control group; SIT, sensory integration training group; EEG, electroencephalogram biofeedback group; MUL, multimodal intervention group.*

*Compared with the control group,*

* *P < 0.01; compared with the psycho-behavioral group,*

& *P < 0.05; compared with the biofeedback group,*

# *P < 0.05*.

**Table 8:** Comparison of hyperactivity index scores before and after treatment in children with hyperactive-impulsive type.

**Groups**	**Number**	**Hyperactivity index**			
		**Pre-intervention**	**Post-intervention**	***t***	***P***
CON	13	1.55 ± 0.39	1.50 ± 0.33	0.46	0.65
SIT	13	1.80 ± 0.54	1.67 ± 0.57	4.12	0.001
EEG	13	1.71 ± 0.56	1.56 ± 0.50	3.83	0.002
MUL	13	1.82 ± 0.51	1.63 ± 0.47^**^,^&^,^#^	5.69	0.000
*F*		0.82	5.27		
*P*		0.49	0.003		

*CON, control group; SIT, sensory integration training group; EEG, electroencephalogram biofeedback group; MUL, multimodal intervention group.*

*Compared with the control group,*

** *P < 0.01; compared with the psycho-behavioral group,*

& *P < 0.05; compared with the biofeedback group,*

# *P < 0.05*.

**Table 9:** Comparison of impulsive-hyperactivity scores before and after treatment in children with complex type.

**Groups**	**Number**	**Impulsive-hyperactive**			
		**Pre-intervention**	**Post-intervention**	***t***	***P***
CON	13	1.95 ± 0.67	1.87 ± 0.51	0.56	0.59
SIT	13	2.08 ± 0.7	1.74 ± 0.65	15.25	0.000
EEG	13	2.16 ± 0.85	1.81 ± 0.69	5.55	0.000
MUL	13	2.09 ± 0.72	1.3 ± 0.6^*^,^&^,^#^	10.63	0.000
*F*		0.21	2.5		
*P*		0.89	0.07		

*CON, control group; SIT, sensory integration training group; EEG, electroencephalo -gram biofeedback group; MUL, multimodal intervention group.*

*Compared with the control group,*

* *P < 0.05; compared with the psycho-behavioral group,*

& *P < 0.05; compared with the biofeedback group,*

# *P < 0.05*.

**Table 10:** Comparison of hyperactivity index scores before and after treatment in children with complex type.

**Groups**	**Number**	**Hyperactivity index**			
		**Pre-intervention**	**Post-intervention**	***t***	***P***
Con	13	1.79 ± 0.59	1.78 ± 0.60	0.26	0.8
SIT	13	2.08 ± 0.74	1.72 ± 0.64	8.3	0.00
EEG	13	2.04 ± 0.66	1.78 ± 0.71	3.04	0.01
MUL	13	1.99 ± 0.61	1.11 ± 0.27^*^,^&^,^#^	5.81	0.00
*F*		0.53	4.41		
*P*		0.66	0.08		

*CON, control group; SIT, sensory integration training group; EEG, electroencephalogram biofeedback group; MUL, multimodal intervention group.*

*Compared with the control group,*

* *P < 0.05; compared with the psycho-behavioral group,*

& *P < 0.05; compared with the biofeedback group,*

# *P < 0.05*.

## Discussion

Non-pharmacological treatment of ADHD has received increasing attention from parents of children with ADHD and domestic and foreign researchers due to its few side effects and small economic burden. The results of this study showed that sensory integration training combined with multimodal intervention of EEG biofeedback therapy could significantly increase the level of concentration in children with ADHD and significantly improve the behavioral symptoms of impulsive-hyperactivity and hyperactivity. Sensory integration training combined with EEG biofeedback therapy multimodal intervention is more effective than single intervention in improving core symptoms in children with ADHD.

### Effect of Sensory Integration Training on the Improvement of Core Symptoms in Children With ADHD

Sensory integration training can improve the hand-eye coordination function, improve the level of attention, coordinate visual hearing, touch, proprioception and vestibular sensation, make the neural pathway unobstructed, so that the children can timely adjust themselves according to the changes in the environment and make adaptive responses, so that the children are emotionally stable, focused and moderately active (33), which is conducive to the children to maintain attention and emotionally tend to be stable, thereby alleviating the children's behavioral problems such as inattention, impulsivity and hyperactivity. Sensory integration training can effectively relieve the symptoms of children with ADHD, improve their attention and self-control ability, and continuously enhance the sensory coordination ability of children, which can not only reduce the symptoms, but also has no side effects (34). Physicians of sensory integration training need to develop perfect and unique training methods according to the actual sensory disorders of children with ADHD, combined with training equipment, so that children can train their various senses in pleasant games, improve self-restraint, achieve the coordinated operation of the body and environment, and effectively improve the symptoms of children (35). Jung and Jung (36) conducted a 3-month sensory-motor integration training (SMI-Tx) intervention in 94 pupils with ADHD aged 7.5–10.1 years, and the results showed that SMI-Tx significantly improved students' ADHD symptoms, specifically, symptoms such as hyperactivity, impulsivity, emotional instability, poor execution, and inattention, significantly increased teacher rating scale (behavioral performance in school life) scores, and level-dependent functional magnetic resonance revealed increased activation in frontal and prefrontal regions, with activation patterns more similar to healthy controls. And the frequency of SMI-Tx treatment, ADHD symptoms improved more significantly. Mojtaba et al. (37) showed that a sensory motor integration training (with an emphasis on Proprioceptive and Vestibular senses) intervention of 2 h/time, twice/week, for 12 weeks resulted in significant improvements in inattention, hyperactivity, and impulsivity scores in ADHD students aged 6–12 years. Studies have shown that the core symptom of children with ADHD is impaired executive ability (38), but executive function training can better control themselves according to environmental changes by coordinating the senses and nerves of children and achieve the effect of improving their executive ability (39, 40). Tamm et al. (41) showed that metacognitive executive function training could significantly improve executive function in children with ADHD, as shown by significantly increased scores in attention, transfer, emotion regulation, working memory and planning, significantly decreased attention deficits, impulsive- hyperactivity scores, and significantly increased visual attention scores. Tamm et al. (42) showed that an 8-week metacognitive executive function training intervention significantly improved executive function (visual/auditory attention, working memory, and cognitive flexibility), correspondingly increased parental scores on executive function, and significantly improved inattention symptoms in children with ADHD, indicating that executive function training is a promising treatment for children with ADHDH. The results of this study showed that a 4-month sensory integration training intervention could significantly increase concentration time and decrease impulsive-hyperactivity and hyperactivity index scores in children with the three types of preschool ADHD compared with those before intervention.

### Improvement of Core Symptoms in Children With ADHD by EEG Biofeedback

Electroencephalogram (EEG) biofeedback has also become neurofeedback (NF), which is a relatively new and reliable method for the treatment of a variety of brain-related diseases, it is also a non-pharmacological treatment for ADHD in children. Through the sensor placed on the scalp to measure brain activity and use the computer to process the frequency signal of brain waves, feed back to the children to let them know the changes of brain waves, change the brain wave pattern through reflex regulation during audiovisual games, change the brain wave waveform persistently through a period of self-regulation, and the patients receive feedback about their own brain activity, thereby improving the self-regulation function of the brain. Many clinical experiments have found that neurofeedback can effectively treat the symptoms of ADHD, improve the patient's attention, reduce ADHD symptoms, reduce impulsive behavior, and improve academic performance and social communication ability (43–45). Duric et al. (46) used a randomized controlled clinical study to evaluate the efficacy of neurofeedback in the treatment of ADHD in children and adolescents, and the results showed that neurofeedback therapy could significantly improve attention disorder and ADHD symptoms in children and adolescents with ADHD, and the effect was as effective as neurostimulant drugs. Geladé et al. (47) performed neurofeedback therapy in children with ADHD and compared the results with excitatory drug therapy and physical activity intervention, and the results showed that neurofeedback therapy significantly improved attention disorders as well as hyperactivity and impulsivity symptoms in children with ADHD, although excitatory drugs were superior to neurofeedback and physical activity in reducing core symptoms in children with ADHD. Maurizio et al. (48) compared the efficacy of EEG neurofeedback and EMG biofeedback in children with ADHD and showed that neurofeedback therapy significantly improved core symptoms in children and adolescents with ADHD, The total score of parent-rated DSM-IV checklist and the scores of attention deficit and impulse/hyperactivity decreased significantly, and the total score of Conners' Parents Rating Scale (DSM-IV), the scores of attention deficit disorder (DSM-IV) and hyperactivity/impulsiveness (DSM-IV) decreased significantly, parents' Strengths and Difficulties Questionnaire hyperactivity scores as well as total problem scores were significantly reduced, Inventory Behavior Rating of Executive Function (teacher and parent) behavior regulation scores were significantly reduced, The Conners' Teacher Rating Scale total index score was significantly reduced. Duric et al. (49) showed that neurofeedback therapy significantly improved core symptoms in children and adolescents with ADHD, as shown by a significant increase in concentration time, a significant decrease in hyperactivity scores, and a significant increase in school performance. Razoki (50) compared neurofeedback with psychostimulants for the treatment of attention deficit children and adolescents and concluded that the role of neurofeedback therapy in the treatment of ADHD in children should be considered as a supplement to multiple treatment modalities, personalized treatment for children's needs, and as a feasible alternative to stimulant drugs for specific patients. Particularly for those patients with mental illness who have low drug response, high baseline theta power spectrum due to unbearable side effects of drug treatment, and possibly no complications would derive more benefit from neurofeedback therapy. The results of this study showed that after 4 months of EEG biofeedback treatment, the concentration time of children with ADHD was significantly increased compared with that before intervention, and the impulsive-hyperactivity and hyperactivity index scores were significantly decreased, consistent with the previous findings.

### Effect of Multimodal Treatment on the Improvement of Core Symptoms in Children With ADHD

Salami et al. (51) conducted a multimodal intervention (based on executive function and sensory integration) for children with attention-deficit/hyperactivity disorder for 8 weeks, 3 times/week, 1.5 h/time, the results showed that combination therapy based on executive function and sensory integration child-centered reduces attention deficit and hyperactivity. Valõe et al. (52) studied the long-term effects of multimodal therapy on psychopathology and health-related quality of life in children with ADHD, and the results showed that 36 months of multimodal therapy significantly improved psychopathology and health-related quality of life in children with ADHD, Specifically, the scores of major depressive episode, dysthymic disorder, (hypo) manic episode, attention deficit/hyperactivity disorder, separation anxiety disorder, social phobia, conduct disorder, oppositional defiance disorder, and generalized anxiety disorder were significantly increased. Rajabi et al. (53) showed that neurofeedback combined with game-based cognitive training improved related symptoms more significantly in children with ADHD than single interventions, as shown by significant increases in visual attention score, total attention score as well as Conners' Parent Rating Scale (attention) score and Conners' Teacher Rating Scale (attention) score, and significant improvements in response control (visual impulsivity) as well as total response control (impulsivity) score as well as Conners' Parent Rating Scale (impulsivity/hyperactivity) score and Conners' Teacher Rating Scale (impulsivity/hyperactivity) score. For the Cz area, there was an increase in the activity (amplitude μV) of beta waves, and SMR wave. Also, there was a decrease in the activity (amplitudeμV) of the θ/β ratio; For the FCz area, there was an increase in the activity (amplitudeμV) of SMR waves. Li et al. (54) studied the efficacy of methylphenidate combined with EEG feedback in the treatment of children with ADHD, and the results showed that patients in the methylphenidate combined with EEG feedback treatment group had significantly improved attention deficit scores, hyperactivity/impulsivity scores, and total scores in the ADHD parent-teacher questionnaire, as well as improved hyperactivity scores in the Conners' parent-teacher questionnaire, significantly reduced peer interaction assessment scale scores, significantly increased global functional assessment scores, and significantly improved brain function compared with the control group using methylphenidate alone. These findings confirm that, regardless of the combined intervention strategy, multimodal intervention is more effective than single intervention in improving symptoms in children with ADHD. In this study, a non-drug combined intervention model was used, and the results revealed that sensory integration training combined with EEG biofeedback therapy could significantly improve the concentration time of children with all types of ADHD, and also improve the impulsive-hyperactivity and hyperactivity index scores of the PSQ scale, and more importantly, the effect of multimodal intervention was better than that of sensory integration training intervention alone or EEG biofeedback therapy intervention. However, the degree of improvement in symptoms and PSQ scores was slightly different in children with different types of ADHD. Therefore, personalized intervention programs should be developed for children with different types of ADHD. At the same time, psycho-behavioral intervention in children with ADHD is a long-term process. Although this study found some improvement in PSQ scale scores in children with ADHD after 4 weeks of intervention, further continuous intervention and follow-up validation are needed to systematically evaluate the long-term efficacy of multimodal intervention treatment.

## Conclusion

Multimodal intervention can significantly improve the concentration level of children with ADHD, and significantly improve the behavioral symptoms of impulsive-hyperactivity and hyperactivity. multimodal interventions were more effective than single interventions in improving core symptoms in children with ADHD. The results of this study provide a reference for related research and practical application.

However, the degree of improvement in symptoms and PSQ scores was slightly different in children with different types of ADHD. Therefore, personalized intervention programs should be developed for children with different types of ADHD. In addition, neurobehavioral intervention in children with ADHD is a long-term process. Although this study found that the core symptoms of children with ADHD were improved to some extent after the intervention, to systematically evaluate the long-term efficacy of comprehensive intervention treatment, further continuous intervention and follow-up verification are needed. In addition, the sample size included in this study was small, and subsequent work needs to expand the sample size to verify these conclusions.

## Data Availability Statement

The raw data supporting the conclusions of this article will be made available by the authors, without undue reservation.

## Ethics Statement

The studies involving human participants were reviewed and approved by Children's Hospital Affiliated to Xi'an Jiaotong University. Written informed consent to participate in this study was provided by the participants' legal guardian/next of kin.

## Author Contributions

KN: software, resources, writing—original draft preparation, experimental design, and writing—review and editing. TW: writing—review and editing, data processing, and experiment. All authors have read and agreed to the published version of the manuscript.

## Funding

This study was supported by 2019 Postdoctoral Phase Research Result of Education Entry Station of Shaanxi Normal University (Entry No.: 230180) and 2020 Ministry of Education Humanities and Social Sciences Research Planning Fund Project (Project Approval No.: 20YJA890019).

## Conflict of Interest

The authors declare that the research was conducted in the absence of any commercial or financial relationships that could be construed as a potential conflict of interest.

## Publisher's Note

All claims expressed in this article are solely those of the authors and do not necessarily represent those of their affiliated organizations, or those of the publisher, the editors and the reviewers. Any product that may be evaluated in this article, or claim that may be made by its manufacturer, is not guaranteed or endorsed by the publisher.
